# BG-4 from Bitter Gourd (*Momordica charantia*) Differentially Affects Inflammation In Vitro and In Vivo

**DOI:** 10.3390/antiox8060175

**Published:** 2019-06-14

**Authors:** Andrea Nieto-Veloza, Zhihong Wang, Qixin Zhong, Hari B. Krishnan, Vermont P. Dia

**Affiliations:** 1 Department of Food Science, University of Tennessee, Knoxville, TN 37996, USA; cnietove@vols.utk.edu (A.N.-V.); zwang78@vols.utk.edu (Z.W.); qzhong@utk.edu (Q.Z.); 2 Agricultural Research Service, United States Department of Agriculture (USDA), Columbia, MO 65211, USA; Hari.Krishnan@ars.usda.gov

**Keywords:** BG-4, bitter gourd, colitis, inflammation, macrophages

## Abstract

BG-4 isolated from bitter gourd has been reported for anti-cancer properties. The objective was to evaluate the anti-inflammatory properties of BG-4 in vitro and in vivo. Comparative study of the anti-inflammatory properties of BG-4 in vitro and in vivo was conducted on lipopolysaccharide (LPS)-activated mouse macrophages, and on dextran sodium sulfate (DSS)-induced colitis in mice. BG-4 reduced the production of pro-inflammatory markers in LPS-activated macrophages. On the other hand, intraperitoneal administration of BG-4 in DSS-induced colitis led to colon shortening, elevated neutrophils infiltration and myeloperoxidase activity, presence of blood in the stool, and loss of body weight, with differential systemic and local effects on pro-inflammatory cytokines in vivo. The results demonstrated that BG-4 differentially affected inflammation in vitro and in vivo.

## 1. Introduction

Inflammatory bowel disease (IBD) is a life-long condition of chronic and relapsing inflammation of the gastrointestinal tract associated with immune-mediated disorders [1]. It has two major phenotypes: Crohn’s disease, which can severely affect any part of the bowel, and ulcerative colitis (UC), which involves damage in the superficial mucosa of the colon, starting always from the anus [1,2]. IBD patients are at major risk of developing gastrointestinal and extra-intestinal carcinomas, especially those carrying UC whose probability for developing colitis-associated colorectal cancer (CACC) increases with the length and severity of inflammatory manifestation [3]. The increasing incidence of UC, especially at a younger age, has raised a global concern considering the detrimental consequences in terms of quality of life, economic loss associated with lower productivity and increased medical expenses, and the imminent risk of development of CACC [2,4]. Hence, alternative ways to manage inflammation are needed, and plant origin compounds may prevent, ameliorate and alleviate chronic inflammation, and consequently the risk of IBD and associated diseases.

*Momordica charantia*, commonly known as bitter gourd, is a member of the Cucurbitaceae family mainly cultivated in Asia, Africa, and South America [5]. It is traditionally consumed as a vegetable and with medicinal purposes such as treatment of inflammation, fever, rheumatism, as well as antidiabetic and anti-helmitic [6]. Several studies have addressed in vitro and/or in vivo effects of the whole edible parts or extracts with demonstrated anti-oxidant, anti-inflammatory, anti-diabetic, anti-cancer and metabolic regulatory properties [7,8,9,10,11,12].

BG-4 is a novel 4 kDa peptide isolated from bitter gourd seeds with trypsin inhibitory and in vitro anti-colon cancer and anti-inflammatory properties [13,14]. BG-4 was isolated from the seeds of *Momordica charantia* by 70% ethanol extraction. It showed very strong trypsin inhibitory activity which is at least 8x more potent than purified soybean Kunitz trypsin inhibitor [13]. Moreover, mass spectrometric analysis showed the following peptide sequence matches: SWPQLVGSTGAAAK, VGSPVTADFR, GIVARPPAIG and DSDCLAQCICVDGHCG [13]. However, the potential bioactive properties of BG-4 in animal models remain unexplored. Herein, we made a comparative in vitro and in vivo anti-inflammatory study of BG-4, by measuring the expression of pro-inflammatory markers in lipopolysaccharide (LPS)-activated mouse-derived macrophages, and in dextran sodium sulfate (DSS)-induced colitis in mice. Our results indicate that BG-4 has differential effects in inflammation within in vitro and in vivo settings.

## 2. Materials and Methods

### 2.1. Extraction of BG-4

The 4 kDa peptide BG-4 was extracted from bitter gourd seeds as described previously [13].

### 2.2. In Vitro Antioxidant Activity of BG-4

The antioxidant capacity of BG-4 was tested via the oxygen radical absorbance capacity assay (ORAC) as described by Vernaza and coworkers [15] with slight modifications. In brief, 150 μL of fluorescein solution prepared in phosphate buffer (75 mM, pH 7.4) were plated in a black 96-well plate. Twenty-five microliters of Trolox standard curve (100–3.125 μM in phosphate buffer), blank or sample diluted in phosphate buffer were added and incubated at 37 °C for 30 min. Then, 25 μL of 2,2′-azobis (2-amidinopropane) dihydrochloride (AAPH) (Sigma-Aldrich, St. Louis, MO, USA) solution at 41.5 mg/mL in phosphate buffer was added and fluorescence was read at 485 nm/20 nm excitation and 528 nm/20 nm emission wavelengths for 2 h every min. Each sample and standard curve point was measured in triplicate and results were expressed as μM of Trolox equivalents/g of BG-4. Radical scavenging activity was measured by quantifying the inhibition of 2,2-diphenyl-1-picrylhydrazyl free radical (DPPH•). Briefly, 100 μL of sample and blank were plated in a clear 96-well plate, then 100 μL of 100 μM DPPH• (Sigma-Aldrich, St. Louis, MO, USA) dissolved in methanol were added and incubated in dark for 30 min. Simultaneously, a combination of sample and methanol, instead DPPH•, were tested as reference for background signal. Absorbance was read at 517 nm. The absorbance of methanol reference was subtracted and the percentage of inhibition was calculated against blank. Results are presented as percentage of scavenging rate of DPPH•.

### 2.3. Measurement of Pro-Inflammatory Markers In Vitro

Murine RAW 264.7 macrophages were cultured in DMEM supplemented with 10% heat-inactivated fetal bovine serum (Life Tech, Carlsbad, CA, USA) and 1% penicillin/streptomycin at 37 °C in a humidified 5% CO_2_ incubator. Cells were seeded at 2 × 10^5^ cells/well in 6-well plates in 2 mL media, or 5 × 10^3^ cells/well in 96-well plates in 200 μL media and allowed to attach overnight. Cells were treated with BG-4 (0–500 μg/mL) for 8 h and stimulated with LPS (1 μg/mL) for 16 h. After which, supernatant was collected for TNF-α and IL-6 measurement via ELISA following the manufacturer’s protocol (BioLegend, San Diego, CA) and nitric oxide (NO) production by Griess reagent assay. Whole cell lysates were collected for immunoblotting of inducible nitric oxidase synthase (iNOS) and cyclooxygenase-2 (COX-2) (ProteinTech, Chicago, IL, USA) by chemiluminescence following standard protocol. Cell viability was tested by MTS assay following the manufacturer’s protocol (Promega, Madison, WI, USA).

### 2.4. Dosage Information and In Vivo Experimental Procedure

The BG-4 dose used in the animal study (15 mg/kg body weight (bw)) is equivalent to the optimum concentration (375 µg/mL) of BG-4 in vitro producing significantly decreased expression of pro-inflammatory markers without affecting cell viability. This translation assumes an average mouse weight of 25 g and circulating blood of 1 mL. This will be equivalent to a daily intake of two 500 mg capsules as a dietary supplement for a 70-kg person. The protocol for the animal experiment was approved by the Institutional Animal Care and Use Committee of the University of Tennessee Knoxville (Approval Protocol #2591-0418) and followed guidelines of the National Institutes of Health guide for the care and use of Laboratory Animals (NIH Publications No. 8023, revised 1978). Twenty-two male C57BL/6 7-week old mice (Jackson Laboratories, Harbor, ME, USA) were randomized and housed in pairs in standard mouse cages with water and food provided ad libitum, under standard controlled conditions (23 ± 2 °C, 30–70% relative humidity with 12 h light and 12 h dark cycle). Mice were randomly divided into three groups and treated as follows: control group (CG, *n* = 6), DSS-treated group (DSS, *n* = 8), and DSS + BG-4-treated group (BG-4, *n* = 8). Mice in CG were administered with normal drinking water, while DSS and BG-4 groups received drinking water with 3% DSS (MW = 36–50 kDa, MP Biomedicals, Santa Ana, CA) to induce colitis. Two stages of DSS administration were performed allowing a recovery period in between, in order to simulate the periods of relapse and remission that IBD patients experience [16]. Daily intraperitoneal injection (IP) of 100 µL sterile water was performed over CG and DSS groups, while BG-4 received IP of 15 mg BG-4/kg bw dissolved in 100 µL sterile water. Food intake and body weight were recorded daily. Presence of visible blood (in stool or anus), as well as stool consistency, were monitored and scored daily. Stool samples were collected from the cages every three days to evaluate the presence of occult blood via quantification of hemoglobin in feces. At day 15, mice were anesthetized with isoflurane, blood collected by cardiac puncture, followed by cervical dislocation. Colon was removed, washed with PBS, length measured and cut longitudinally into two pieces: one for hematoxylin and eosin (H&E) staining, and the other frozen in liquid nitrogen for biochemical analysis.

### 2.5. Myeloperoxidase Assay in the Colon

Neutrophils infiltration was assessed by measuring myeloperoxidase (MPO) activity in colonic extracts as reported previously [17] with slight modifications. Colonic extracts were obtained by beads homogenization of 50 mg of colon samples with 1 mL buffer (5 g of hexadecyltrimethylammonium in 1 L of 50 mM potassium phosphate buffer at pH 6.0), followed by centrifugation at 20,000× *g* for 15 min at 4 °C. Supernatant was collected and further centrifuged to ensure total precipitation of solid tissue. Ten µL of supernatant was plated in triplicate in a 96-well plate and combined with 200 µL freshly prepared *o*-dianisidine solution (16.7 mg of o-dianisidine, 90 mL of deionized water and 10 mL of potassium phosphate buffer, combined with 50 µL of 1.2% H_2_O_2_). Absorbance at 450 nm was recorded every 30 s for 5 min. MPO activity was calculated as the amount needed to degrade 1 µmol H_2_O_2_/min at 21–22 °C per mg protein in the colonic extract.

### 2.6. Measurement of Hemoglobin Content in the Feces

Presence of occult blood in the feces was assessed by measuring hemoglobin content [18,19]. Twenty mg of freeze-dried pulverized samples were vortexed with 100 µL dH_2_O, and boiled for 10 min. Six hundred microliters of 30% acetic acid was added, vortexed for 2 min, then 900 μL of ethyl acetate was added, and organic layer was collected in a separate microcentrifuge tube after centrifugation at 2000× *g* for 3 min. In a quartz cuvette, equal parts of the organic phase and TMB solution (14.4 mg of 3,3′,5,5′-tetramethylbenzidine in 100 mL mixture of glacial acetic acid/dH_2_O/ethanol 20/30/50) were mixed and the reaction was started by adding the same amount of 3% H_2_O_2_. The absorbance was recorded at 660 nm at 30 s, 60 s, and 90 s. Hemoglobin content was calculated as the average for the three time points per mg of feces using bovine hemoglobin (Alfa Aesar, Ward Hill, MA, USA) standard curve.

### 2.7. Measurement of Cytokines by ELISA

Supernatants of treated macrophages, as well as blood serum and colonic extracts, were used to measure the expression of pro-inflammatory cytokines using ELISA kits according to manufacturer instructions (BioLegend, San Diego, CA, USA).

### 2.8. Statistical Analysis

All experiments were performed in triplicate. Results were reported as mean ± SD or SE. Analysis of variance (ANOVA) and Tukey’s test were used to establish significant differences (*p* < 0.05) and *t*-student for comparison of two samples or groups, using Statgraphics Centurion ^®^ software (Statgraphics Inc, The Plains, VA, USA).

## 3. Results and Discussion

### 3.1. BG-4 Exert Antioxidant and Antiradical Activity

**Figure 1:**
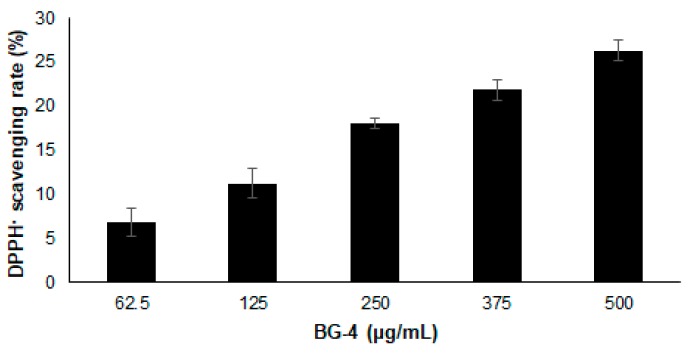
Radical scavenging activity of BG-4 calculated as % of inhibition of DPPH free radical for different concentrations. Results are presented as mean ± SD.

Antioxidant capacity of BG-4 was estimated as 4.72 ± 0.41 μM of Trolox Equivalent per gram, being comparably higher to that reported for protocatechuic acid and protocatechuates (<3 μM of Trolox Equivalent/g) [20]. The ability of BG-4 to convert DPPH radical into it picrylhydrazine form increase about 1.6 times when doubling the concentration, as it can be seen in Figure 1, exerting up to 26% inhibition at a concentration of 0.5 mg/mL of BG-4.

### 3.2. BG-4 Decreased Expression of Pro-Inflammatory Markers In Vitro

**Figure 2:**
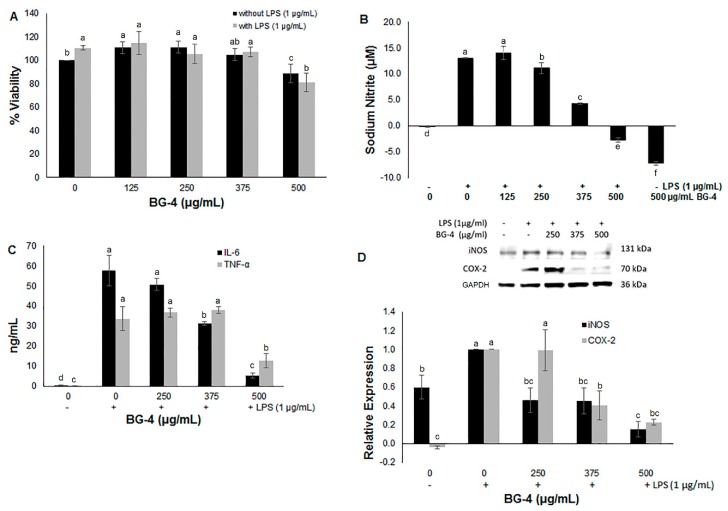
Effect of BG-4 on viability of activated lipopolysaccharide (LPS+) and non-activated (LPS-) macrophages (**A**), NO production (**B**), cytokine production (**C**), and iNOS and COX-2 expression (**D**), in vitro. Results are presented as mean ± SD for (**A**–**C**) and mean ± SE for (**D**). Different letters indicate significant differences (*p* < 0.05) among treatments.

Macrophages are part of the first line of defense against injury, mediating the innate non-specific immune response via inflammation, and playing an important role not only in host defense but in tissue homeostasis, repair, and pathology development including IBD [21,22]. RAW 264.7 mouse derived macrophages have been widely used as in vitro model to study modulatory effects of several compounds using LPS for activation [23,24,25]. Treatment with BG-4 at concentrations up to 375 µg/mL did not affect the viability of macrophages (Figure 2A). It is known that LPS can activate NF-κB which acts as a master regulator of the inflammatory response by promoting the release of signaling and effector molecules such as pro-inflammatory cytokines (IL-6, TNF-α, IL-1β) and nitric oxide (NO) that act as mediators of inflammation during the host immune response [6]. The destruction of normal and healthy tissue as a result of chronic inflammatory condition can lead to IBD, rheumatoid arthritis and multiple sclerosis [26], hence reducing the level of these pro-inflammatory secreted molecules can lead to management and alleviation of these diseases. BG-4 dose-dependently reduced the production of NO (Figure 2B) and IL-6 (Figure 2C) in LPS-activated RAW 264.7 macrophages. Moreover, the expression of iNOS and COX-2 (Figure 2D) was reduced by BG-4.

**Figure 3:**
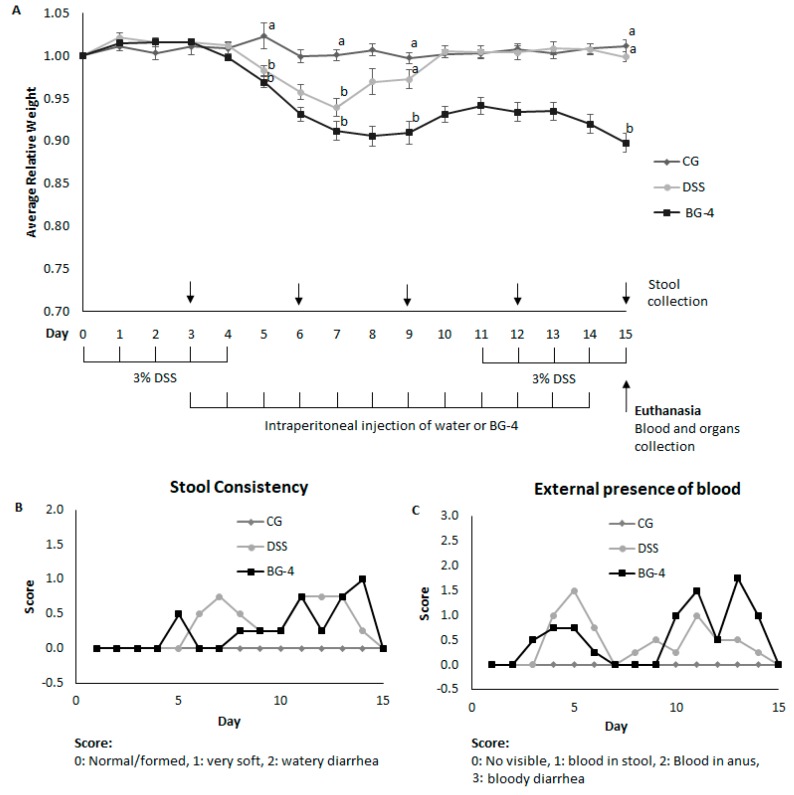
Animal study design and effect of BG-4 treatment over relative weight (**A**), stool consistency (**B**), and presence of visible blood (**C**) on dextran sodium sulfate (DSS)-induced colitis in mice. Scores for stool consistency and presence of visible blood for CG were 0 for every observation. Results are presented as mean ± SE. Different letters indicate significant differences (*p* < 0.05) among treatments.

These results suggest that the mechanistic effect of BG-4 had a major impact on molecules particularly involved in the pathway associated with the specific expression of iNOS and IL-6, and consequently over their downstream products, but affected in a lower extent the expression of COX-2 and TNF-α. Hence, we selected 375 µg/mL concentration to study the ability of BG-4 to ameliorate DSS-induced colitis in mice with a translation of IP administration of BG-4 at 15 mg/kg bw assuming an average mouse weight of 25 g and a circulating blood volume of one milliliter. Two stages of DSS administration were performed in order to induce damage to the epithelial barrier in the colon to simulate colitis, allowing a period of recovery in between as presented in Figure 3A. This experimental design mimics the periods of remission and relapse that IBD patients usually experience [16]. BG-4 administration started at three days post DSS to mimic ingestion of BG-4 as a supplement for ameliorating established IBD (versus prevention) in human as there are no known risk factors to IBD. Hence, it is highly likely that dietary supplement will be consumed for management and not for the prevention of IBD.

### 3.3. IP Administration of BG-4 did not Alleviate Indicators of Colitis in Vivo

The damage in the colonic mucosa that occurs in ulcerative colitis results in different symptoms and indicators such as diarrhea, presence of blood in the stool, increased frequency of bowel movements, mucus discharge, abdominal pain, weight loss, bloody diarrhea, and rectal bleeding irritation and fissures [27]. Daily monitoring of body weight, presence of visible blood and stool consistency were performed, in order to establish variation of external indicators of the disease. CG maintained a constant incremental increase in weight while the DSS group presented a significant decrease starting at day five, and minimum trough at day seven, with a 6% loss of original weight. BG-4 administration in DSS-treated mice showed a continued loss in weight until day nine and in days 14 and 15 with a 10% decrease in body weight at euthanasia as shown in Figure 3A. For CG, no visible blood in the stool or anus and normal stool consistency were observed with a disease score of zero (Figure 3B) neither DSS nor BG-4 groups presented watery or bloody diarrhea, but changed in stool consistency, becoming much softer in comparison to CG group and BG-4 administration led to stool softening earlier than DSS group as shown in Figure 3B. Presence of blood in stool was observed for DSS and BG-4 groups mostly between days 3–6 and 10–14, but the frequency of mice presenting blood in the anus was higher for BG-4 than for DSS group as presented in Figure 3C.

Overall, external indicators of the disease suggest that DSS administration induced colitis with a mild grade of severity in this study. Since diarrhea or reduction in food intake were not observed, loss of body weight might be due to an increased frequency in bowel movements with a softened stool consistency and presence of blood, that upon removal of DSS was compensated by food intake, allowing partial or total recovery of the animals. However, the lower extent of recovery for BG-4 group, in addition to the higher frequency of animals with rectal bleeding may indicate that IP administration of BG-4 aggravated symptoms of the disease.

### 3.4. IP Administration of BG-4 Aggravated DSS-Induced Colitis in Mice

**Figure 4:**
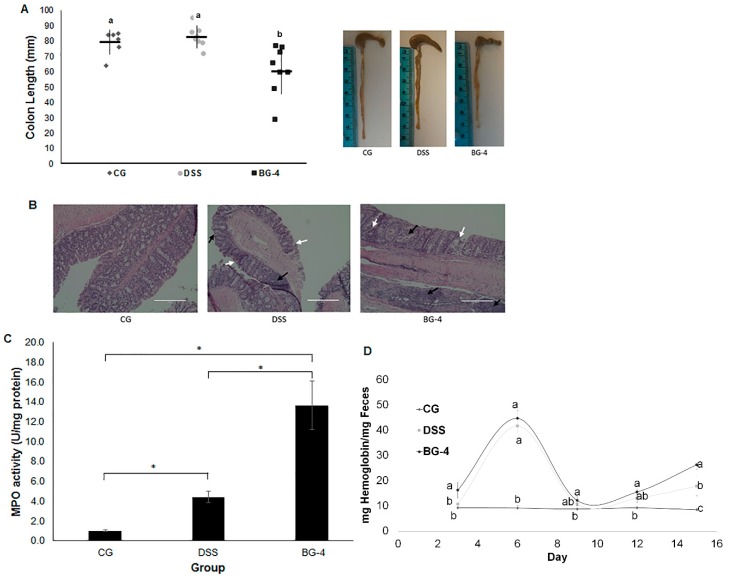
Colon length (**A**), H&E staining of colonic tissue (**B**), MPO activity (**C**), and fecal hemoglobin (**D**) on DSS-induced colitis in mice. Results are presented as mean ± SD (**A**) and (**C**) or SE (**D**). Different letters and * indicate significant differences (*p* < 0.05) among treatments. Immune cells infiltration and distortion of the crypt architecture are indicated by black and white arrows, respectively.

Colon shortening has been reported for several studies as an outcome of colitis in animal models [6,28,29,30]. BG-4 group presented a significant reduction in average colon length of about 2 cm in comparison with CG as shown in Figure 4A. H&E staining allows a comparison of the effects of DSS challenge and BG-4 treatment over the internal structure of colonic tissue. Micrographs of stained colonic tissue for the three groups show that animals under DSS-induced colitis exhibited immune cells infiltration (black arrows) in the lamina propria, and a distortion of crypts architecture (white arrows) accompanied by the loss of goblet cells (Figure 4B). In the case of BG-4, infiltration was extended to the muscularis region and to form crypt abscesses and reach the glandular lumen of the crypts forming abscesses as shown in Figure 4B.

MPO is a peroxidase enzyme that plays an important role during infections by modulating the production of hypochlorous acid and other non-selective reactive species that mediate pathogens killing, and can induce host tissue damage by promoting inflammation [31]. It is mainly present in specialized granules of neutrophils hence elevated MPO levels and neutrophil infiltration have been correlated to inflammatory autoimmune disorders [32]. DSS and BG-4 groups exhibited an MPO activity four and 14 times higher, respectively, than CG (Figure 4C), corroborating observations in H&E staining micrographs where a higher infiltration of immune cells was detected for both groups with elevated severity upon BG-4 treatment.

Quantification of hemoglobin in collected feces was used to estimate fecal occult blood. Figure 4D indicates the presence of hemoglobin in feces for BG-4 and DSS groups followed the same trend as loss in weight. At the end of the study, the presence of hemoglobin in feces from BG-4 group was significantly higher than in DSS group, being consistent with the observations and scoring for the visual presence of blood in stool or rectum presented in Figure 3B,C.

### 3.5. IP Administration of BG-4 Exerted a Differential Systemic and Localized Effect in Pro-Inflammatory Cytokines In Vivo

**Figure 5:**
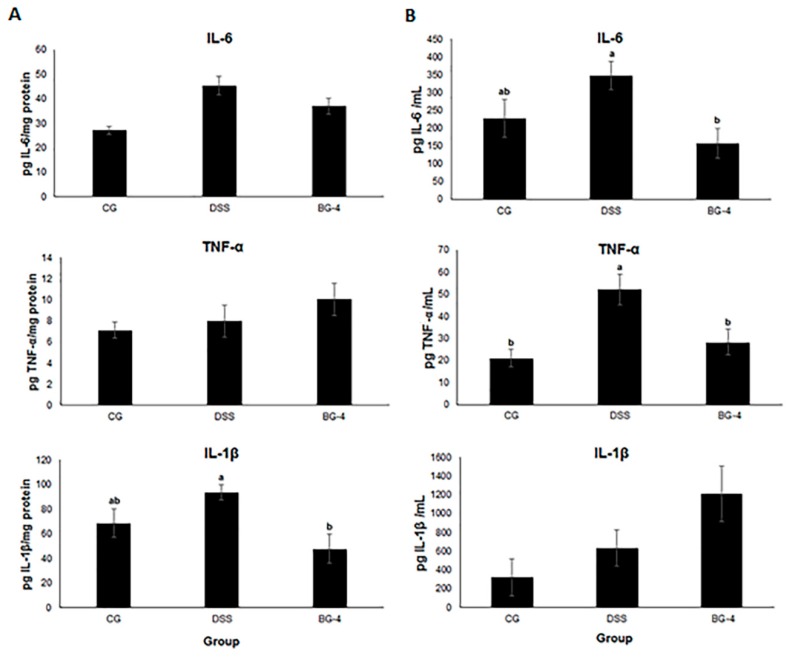
Effect of BG-4 on the level of pro-inflammatory cytokines in colonic extract (**A**) and serum (**B**). Results are presented as mean ± SE. Different letters indicate significant differences (*p* < 0.05) among treatments.

Cytokines are signaling proteins that contribute to the modulation of the immune and inflammatory response during tissue damage and repair. The levels of pro-inflammatory cytokines IL-6, TNF-α, and IL-1β in colonic extracts (A) and in serum (B) are presented in Figure 5. No significant effect was observed upon DSS challenge and/or BG-4 treatment over the expression of IL-6 and TNF-α in colonic extracts, but both compounds were upregulated in serum in the DSS group and attenuated under BG-4 treatment (Figure 5A,B). IL-1β, in contrast, was upregulated in the DSS group and attenuated in the BG-4 group in colonic extracts but remained high in serum (Figure 5A,B). These results suggest that DSS and BG-4 were capable to exert a systemic (in circulating blood) but not localized (in colonic tissue) effect. A recent study has demonstrated dysregulation of pro-inflammatory cytokines in DSS-induced colitis mouse model with similar differential outcomes at the serum and colonic level observed in our study [33]. The particular differences in the systemic and localized effect over cytokines seem to indicate that IP injection allowed BG-4 to reach blood circulation, as expected for the high bioavailability reported for this administration route but post the question if the appropriate concentration was, in fact, capable to reach the intestinal barrier, where the tissue damage was induced via DSS and the inflammatory response was concentrated.

Our results showed that BG-4 exerted a differential effect over inflammation in vitro and in vivo. In LPS-stimulated macrophages, treatment with BG-4 showed a dose-dependent reduction of NO, IL-6, COX-2, and iNOS, suggesting 375 μg/mL as an optimal concentration for significant reduction of inflammatory markers. However, IP administration of BG-4 in a DSS-induced colitis mouse model, aggravated disease symptoms when compared with untreated mice, accompanied by the dysregulation of pro-inflammatory cytokines. The systemic circulation of BG-4 due to IP administration involved a possible effect over other immune and epithelial cells that can be more or less sensitive, in terms of cytotoxicity [34] and alteration of signaling and regulatory pathways, than the tested macrophages. For instance, it has been recently demonstrated the induction of NO metabolism in enterocytes alleviates colitis and CACC [29], suggesting a controversial role of NO in the regulation of the inflammatory response associated with its origin. According to this perspective, suppression of NO production exerted by BG-4 could result in a counteracting effect by simultaneously suppressing NO production on macrophages and enterocytes. 

The outcomes observed in vivo under BG-4 treatment suggest that the mechanistic effect by which BG-4 suppresses NO production may be associated with enterocyte than macrophages. Under this circumstance, the reduced expression of NO by the enterocyte allows neutrophils infiltration, generation of cytotoxic compounds by MPO, tissue damage, and a consequently increased inflammatory response. One strength of our study is the first report on the comparative anti-inflammatory properties of BG-4 peptide from *Momordica charantia* using both in vitro and in vivo models. In addition, both mouse cell line and mouse model of colitis were utilized in the study. On the other hand, the observed contrasting result of in vitro and in vivo models on the anti-inflammatory effect of BG-4 is a potential weakness. The intraperitoneal injection of BG-4 may have resulted in the toxic effect of BG-4 in mouse model resulting in its pro-inflammatory effect which is contradictory in the observed anti-inflammatory effect in vitro. Hence the next step on the potential application of BG-4 for management of inflammatory diseases must be observed via oral route to determine if it can be used as a dietary supplement to mitigate diseases such as colitis.

## 4. Conclusions

In RAW 264.7 macrophages, BG-4 reduced the production of pro-inflammatory molecules NO, IL-6 and TNF-α as well as the expression of COX-2 and iNOS. On the other hand, in DSS-induced colitis mouse model intraperitoneal administration of BG-4 led to diarrhea, blood in stool and loss in weight. Our results demonstrated that IP administration of BG-4 in DSS-induced colitis mouse model led to a worsened outcome of the disease, in contrast to the reduction of the pro-inflammatory markers observed in vitro. BG-4 dose and route of administration must be further studied to realize the role of BG-4 in ameliorating IBD.
